# Grand challenges in analytical chemistry: towards more bright eyes for scientific research, social events and human health

**DOI:** 10.3389/fchem.2013.00005

**Published:** 2013-03-26

**Authors:** Huangxian Ju

**Affiliations:** State Key Laboratory of Analytical Chemistry for Life Science, Department of Chemistry, Nanjing UniversityNanjing, PR China

Analytical chemistry is a measuring science to probe the compositions and structures of matters, study the chemical constituents, contents, distribution, and interaction of matters, and reveal the space-time rules of matter changes. As the eyes of fundamental scientific research and social activities relative to human security and health, the goals of analytical chemistry are to design sensitive, selective and specific detection strategies, measuring principles and analytical methodologies, and develop modern detection devices and instruments as well as relative softwares or control systems. It has played vital roles in discipline frontiers and social events, and provided important supports for the development of life science, material science, energy science, environment science, and space science. Its significance has been shown in different fields such as new drug development, disease diagnosis and early warning, life process study, food and environment safety, quality control of products, economic, and trade, space exploration, forensic medicine, and even anti-terrorist, etc. Currently analytical chemistry has integrated multidisciplinary research achievements to form itself theory systems and new frontier research fields on fundamental levels, while the development of other related disciplines and the progress of human society constantly challenge analytical chemistry to higher needs, which promotes the continuous development of analytical methodologies and detection instruments.

Different from other chemical disciplines, the progress of analytical chemistry mainly benefits from the achievements in other frontier fields. For example, the separation and analysis in micro/nano-scale depend on the early development and successful application of nanotechnology and microfluidics, which are directly related to and benefits from the frontier study of life science, such as genomics and proteomics. The limit of mass and size in space exploration and *in vivo* analysis is also a catalyst for micro/nano-scaled analytical methodologies and detection devices. Another example is the development of instrumental analysis, which first benefits from the achievements in physics such as electromagnetics, optics, mechanics and electrics, material science, and computer, meanwhile, the needs in life science, environment science, and space science hasten new methodologies and detection technologies. Thus, analytical chemistry is established in multidisciplinary development and has the distinct features and outstanding sense of the times. This article focuses on the development and challenges of instrumental analysis including chromatography and micro/nanofluidic analysis, spectroscopic analysis, electrochemical analysis, mass spectral analysis, and imaging detection. Their future perspectives and needs are also discussed.

## Chromatography and micro/nanofluidic analysis

Gas chromatography has been well-developed and used for separation of small organic molecules in different fields such as petrochemical industry, environmental analysis, and food industry, while liquid chromatography and capillary electrophoresis are quickly developed and have been used together with other detection technologies including mass spectral analysis for biomarker detection and protein analysis. The progress of gas chromatography mainly includes the miniaturization of instruments (Lee et al., 2008), the stationary phases (Gu and Yan, 2010), and detectors (Li et al., 2011). The application of new nanomaterials, sub-micromaterials, and functionalized recognition or affinity materials in the preparation of stationary phase or monolithic column is one of the most prominent progresses in this field. The development of multidimensional separation technologies such as two-dimensional gas chromatography, two-dimensional liquid chromatography (Geng et al., 2009), and coupling of gas chromatography and liquid chromatography (Hyötyläinen and Riekkola, 2003) or liquid chromatography and capillary (microchip) electrophoresis (Yang et al., 2003) is also an important achievement. New pretreatment and separation techniques of complex samples including solid-phase microextraction using nanomaterials in fiber coating, high-throughput pretreatment for proteomic, metabonomic, and metallomic analysis and on-line enzymatic reactor techniques have shown their crucial roles.

The unique properties (e.g., large surface area, and remarkable thermal, mechanical, and chemical stability) of nanostructured materials such as carbon-based nanomaterials, silica-based nanomaterials, polymers, and metal nanoparticles have led to their application as desirable coatings in solid-phase microextraction (Mehdinia and Aziz-Zanjani, 2013). The carbon-based nanomaterials include carbon nanotubes, grapheme, fullerenes, organically modified carbon nanomaterials. The unique selectivities of silica-hydride-based stationary for separation of polar and non-polar compounds (Pesek et al., 2013) and boronate-based monolithic column for *Cis*-diol-containing biomolecules such as carbohydrates, glycoproteins, RNA, and nucleosides (Li and Liu, 2012) have extended the application of affinity chromatography as a tool for specific isolation, enrichment, and detection. The chiral stationary phases for enantioseparation have been widely used for resolution and preparation of biochemicals (Tang et al., 2012). The functionlization of column-packing materials and inner wall of separation channel using biorecognition elements such as nucleic acids, antibody/antigen, protein receptors, peptides, lectins and integrin, and bionic recognition materials such as molecularly imprinted polymer (Qu et al., 2009) and nucleic-acid aptamers (Zhao et al., 2012) further promotes the development of affinity chromatography.

The development and throughput of micro/nanofluidic analysis depend on both the manufacture technique of micro/nanostructures including the separation channel, nanoholes, nanowires, and nanoparticles and the progress in related detection instrument and sampling system. A simple avenue is to use directly a capillary as separation microchannel, which can conveniently be coupled with a fracture sampling technique and amperometric microdetector, and shows high separation efficiency for different kinds of analytes (Zhai et al., 2007). Although the preparation and modification of nanoholes are highly challenging, they have been used and shown promising potential in DNA analysis and protein detection.

## Spectroscopic analysis

Illuminant or optical source, optical splitter, and optical detector are three key components in spectroscopic instruments. The appearance of superstrong optical source, ultrahighly resolved optical splitter, and highly sensitive detector as well as the development of optical fiber technique, plasma technique, and nanotechnology during last decade provides important support for the progress of spectroscopic analysis. The miniaturization and high efficiency of these components are the important research topic in this field. For example, a dielectric barrier discharge based microplasma source has been proposed for atomic emission spectrometric analysis of mercury element (Zhu et al., 2008), and a liquid-core waveguide absorption detection method has been presented for nano-liter-scale samples (Pan et al., 2010). Due to the low damage to analyte, particularly biological samples, long excitation, or detection wavelength is attractive (Radziemski et al., 2007). Thus, near-infrared spectroscopy has been extensively studied. The coupling techniques of different spectrometric detection methods with chromatography, electrophoresis, or/and sample pretreatment have been quickly developed.

Based on the new measuring principles, a lot of spectroscopic techniques have been proposed for analysis of inorganic, organic, and biological samples by combining newly emerging signal probes. The absorption and Rayleigh-scattering effects of the surface-plasmon resonance of Au/Ag nanoparticles, specifically, the color associated with these nanoparticles have been utilized for protein and DNA assays (Liang et al., 2012). The resonance energy transfer from donor to acceptor molecule has promoted the development of chemiluminescence analysis (Huang and Ren, 2012), fluorescent detection (Dong et al., 2010), and electrochemiluminescence methodology (Liu et al., 2007). Fast two-dimensional infrared spectroscopy has been developed for probing biomolecule structure and function (Hunt, 2009). To improve the sensitivity of spectroscopic analysis, resonance Raman spectroscopy (Robert, 2009), shell-isolated nanoparticles-enhanced Raman spectroscopy (Li et al., 2010), surface-enhanced Raman spectroscopy (Gao et al., 2012), and surface-enhanced infrared spectroscopy have been proposed. A single-molecule surface and tip-enhanced Raman spectroscopic method has been achieved for single molecule detection (Pettinger, 2010). The non-linear process during the interaction between matter and light at high power density has led to different non-linear spectroscopic methods.

Another challenge in spectroscopic analysis is the development of optical probes. General organic molecules are continuously concerned. The small molecular fluorescent probes have been extensively used in fluorescent detection (Goncalves, 2009) and imaging (Kobayashi et al., 2010) by labeling them to biomolecules. The fluorescent probes with the features of fast response, high sensitivity, good specificity, large Stokes shift, great photostability, high quantum yield, good size-tunability and sometimes long excitation, and emission wavelength up to near-infrared ranges are urgently needed. The modification of fluorescent probe structures for improving their stability and penetrativity, increasing the Stokes shift, and adapting the demand of biological labeling has become the hotspot. The fluorescent features of nanoparticles such as quantum dots can meet the needs for long-time fluorescent tracing of living cells and tissues and analysis of biological samples. The labeling or conjugation of optical nanomaterials to biorecognition elements such as antibody, DNA, and aptamer (Yuan et al., 2012) have been extensively applied in bioanalysis by fluorescence, colorimetry, Raman scattering spectroscopy, surface-plasmon resonance, chemiluminescence, and electrochemiluminescence. Single molecule and single cell analysis has become a frontier research field by using fluorescence correlation spectroscopy and microscopic technologies. *In situ* scanometric assay (Ding et al., 2010), chemiluminescent imaging (Zong et al., 2012), and fluorescent imaging (Chen et al., 2013) have been coupled with signal amplification strategies using multifunctional nanoprobes for sensitive detection of biomolecules. Spectroscopic imaging using small molecules, fluorescent proteins, and luminescent nanomaterials as probes remains a high degree of challenge in both *in vitro* (Wang et al., 2012) and *in vivo* (Dong et al., 2012). Modern optical techniques provide a bright outlook for cell analysis and life processes (Guo et al., 2013).

## Electrochemical analysis

Electrochemical analysis possesses outstanding advantages of fast response, moderate cost, instrumental simplicity, and portability due to easy miniaturization. Since 1960's it has been successively developed each decade from polarography to solid electrode and spectroscopic electrochemistry in 1970's, chemically modified electrodes and microelectrodes in 1980's, electrochemical bioanalysis and biosensors in1990's and nanoelectroanalytical chemistry in last 10 years. Modern electrochemical methods are sensitive, selective, rapid, and facile techniques applicable to biomedical fields, and indeed in most areas of analytical chemistry. The current frontier research topics in electrochemical analysis is to combine electrochemical detection techniques with nanotechnology, biotechnology, and other signal amplification strategies for obtaining the signals in life processes, achieving dynamic, *in situ*, on-line, and resolved monitoring of different species, probing the rules in interaction between molecules and molecular recognition, and developing new electrochemical sensing and detection methods and technologies, including electrochemical imaging technology (Xue et al., 2010) and photoelectrochemical detection (Tu et al., 2010a; Yao et al., 2013).

The application of new materials, particularly nanostructure materials, in electroanalytical chemistry exhibits greatly improved analytical capacities, which leads to new electrochemical sensing disciplines at the interface of chemistry and the life sciences and offers a broad palette of opportunities for researchers. The early introduction of nanotechnology in electrochemical analysis can be traced back to the work published in last century (Xiao et al., 1999). Up to now, several metal nanoparticles such as Pt, Au, Ag, and palladium (Leng et al., 2011) nanoparticles and different carbon-based nanomaterials such as carbon nanotubes (Lou et al., 2001), graphene (Wang et al., 2009), carbon nanohorns (Tu et al., 2009), carbon nanotubes forest, carbon nanofiber (Wu et al., 2007a), nitrogen-doped carbon nanotubes (Tu et al., 2010b), as well as emzyme-functionalized carbon nanomaterials (Lai et al., 2009), and silica-carbon nanocomposite (Wu et al., 2007b) have been the emerging materials for enhancing the electrochemical signal. Some oxide nanoparticles such as TiO_2_, zironium dioxide (Liu et al., 2004), and mesoporous silica (Dai et al., 2004) have also been used for immobilization of proteins to achieve highly sensitive electrochemical biosensing. The high surface-to-volume ratio, high electrical conductivity, chemical stability, biocompatibility, robust mechanical strength, and good catalytic characters of nanomaterials greatly improve the performance of electrochemical detection. Some nanoparticles such as quantum dots can be used as labels for photocurrent and electrochemiluminescent measurements (Zou and Ju, 2004; Schubert et al., 2010). Moreover, further amplification is feasible by creating a multilayered network on the electrode surface (Bertoncello and Forster, 2009).

In electrochemical sensing, immunosensors (Chen et al., 2006), genic sensors (Ye and Ju, 2005; Paleček and Bartošík, 2012), and cytosensors (Du et al., 2005a; Cheng et al., 2009) have become the hot topics. The impedance technique presents impressive potentialities for label-free detection of targets (Katz and Willner, 2003; Daniels and Pourmand, 2007; Giroud et al., 2009). The electrochemical techniques can be used for the studies of tumor cell adhesion and viability (Du et al., 2005b). The nucleic-acid aptamers have easily been used for electrochemical detection of proteins, DNA, metal ions, and small biomolecules with excellent sensitivity and specificity (Swensen et al., 2009; Liu et al., 2010a). Nanomaterials can be used as the carriers of signaling molecules such as redox active molecules and enzymes, and directly used as signal tags for electrochemical biosensing by amperometry, stripping voltammetry (Cheng et al., 2010), electrochemiluminescence (Deng and Ju, 2013), and their enzyme mimetic functions, which leads to a series of signal amplification strategies for voltammetric, impedance, capacitance, electrochemiluminescent, and photoelectrochemical analysis.

## Mass spectral analysis

Upon the successive innovation in ionization technique and mass analyzer and the steady improvement of the association technique with separation techniques, mass spectral analysis has been quickly developed. It has been used for the detection from small molecules to macromolecules such as proteins and DNA, which promotes the development of whole life science, such as genomics, metabonomics, and proteomics. The development of mass spectrometers, including highly efficient ionization techniques, highly resolved, and sensitive mass analyzers with wide mass range, and the series connection of multiplex mass spectrometers has attracted extensive attention. Due to the ability to detect short-lived reaction intermediates or labile metabolites, time-resolved mass spectrometric measurement has become an enabling tool for studies of chemical reactions, chemical kinetics, and biochemical dynamics (Chen and Urban, 2013). New ambient mass spectrometric techniques and the intelligentialized, miniaturized, and multi-functional mass spectrometers with high sensitivity, simple manipulation, fast response, and good specificity have been the urgent demends.

Mass spectrometric sampling under ambient conditions is a viable technique (Takats et al., 2004), which has been continuously developed (Cooks et al., 2006) in electrospray-assisted laser desorption ionization, electrospray ionization (Chen et al., 2007), and electrospray ionization quadrupole time-of-flight mass spectrometry. A versatile ion source for the analysis of materials in open air under ambient conditions (Robert et al., 2005) and an electrostatic axially harmonic orbital trapping (Alexander, 2000) have been proposed for mass spectral analysis. To simplify the detection process and extending the analytes, an extractive electrospray ionization method without need of sample pretreatment (Li et al., 2009), a nanoextractive electrospray ionization technique (Hu et al., 2010) and a direct infusion nanoelectrospray ionization strategy (Erve et al., 2008) have also been presented. The application of laser-ablation inductively coupled plasma/mass spectrometry has been further developed in different fields including chemical analysis of forensic evidence (Orellana et al., 2013).

Matrix-assisted laser desorption/ionization time-of-flight mass spectrometry (MALDI-TOF MS) has become a powerful tool in the analysis of high molecular mass species such as proteins, DNA/RNA, polysaccharides, and synthetic polymers. In order to avoid the high background interference signal in the low-mass region (<700 Da) produced from conventional matrix, surface-assisted laser desorption/ionization time-of-flight mass spectrometry has been developed to eliminate the interference of matrix ion and improve sample homogeneity by using titania particles, porous silicon, ionic liquid, and carbon-based materials (Ma et al., 2013) as the matrix due to their good flexibility for different samples, high surface area and energy transfer efficiency, which is becoming an urgent topic for extending the application of MALDI-TOF MS. To improve the specificity of this assay technique, some affinity reagents such as antibody and aptamer have been conjugated to porous silicon, grapheme, and carbon nanohorns for affinity capture of target from complex samples, respectively. This research project is just making progress.

Mass spectrometric imaging, which can detect hundreds of (unknown) compounds simultaneously in one molecular imaging experiment without need of target-specific labeling, allows analysis and visualization of peptides, proteins, lipids, metabolites, and pharmaceuticals directly from biological tissues and cell samples (Chughtai and Heeren, 2010). It can provide relatively unbiased molecular information in an anatomical context and has been used for drug evaluation, protein identification, proteomic analysis, *in situ* pharmacometabolomes (Sugiura and Setou, 2010), diagnosis, prognosis, and biomarker discovery. Introduced in 1997, MALDI is the most widely used ionization technique for mass spectrometric imaging by using a time-of-flight mass analyzer. Desorption electrospray ionization (Esquenazi et al., 2009) and low-temperature plasma probe (Liu et al., 2010b) have also been used for mass spectrometric imaging. To study more complex biological problems, there will be an increasing need for (bioinformatic) tools. Ongoing efforts to embed mass spectrometric imaging into the interdisciplinary world of life sciences will move the field into the next decade (Mascini and Heeren, 2012).

## Prospective

Probing the composition, content, and structure of matter in time and space needs accurate, sensitive, selective, stable, fast, automated, high-throughput, and even *in situ* analytical methods and protocols. These features are also the eternal goals and challenges of analytical chemistry. The demands of scientific research, social events, and human health are the force driving the development of analytical chemistry, which makes the methods and technologies be continuing without end. The survey of the information and data of matters at extreme conditions such as superhigh temperature, superlow temperature, high pressure, high velocity, strong radiation, and vacuum elevates the levels of analytical chemistry. The new scientific breakthrough and achievements will continuously bring measuring principles and analytical methodologies. In 21st century, life science is the frontier of scientific research, while human development is decided by environmental and food safety. Analytical chemistry will center on these key fields to develop the detection probes, methods, and instruments. It must cater for the new needs in life science research to develop high-through screening strategies, *in vivo* analytical methods, and ultrahigh sensitive even single-molecule detection and imaging technologies, and follow the social demands in urgent events, environmental contamination accidents, and deleterious food to provide new tools for obtaining pollutant information. Thus, analytical chemistry should forwardly introduce new physical concepts and technologies and combine computer, nanotechnology and biotechnology to create new analytical principles, design-automated detection procedures and specific detection probes, and develop new measurement instruments.
